# The Psychometric Properties of the Frost Multidimensional Perfectionism Scale – Brief

**DOI:** 10.3389/fpsyg.2020.01860

**Published:** 2020-08-07

**Authors:** Vivian Woodfin, Per-Einar Binder, Helge Molde

**Affiliations:** Department of Clinical Psychology, Faculty of Psychology, University of Bergen, Bergen, Norway

**Keywords:** perfectionism, FMPS-B, strivings, evaluative concerns, validation, bifactor modeling

## Abstract

Previous psychometric analyses of the Frost Multidimensional Perfectionism Scale and the abbreviated version (FMPS–Brief) have resulted in inconsistent findings regarding the scale’s bidimensionality or unidimensionality. Different studies evaluating the scale with different statistical analyses and comparative samples report different results and recommendations. This study assessed the FMPS-B’s psychometric properties by conducting both confirmatory factor analysis (CFA) and pure bifactor modeling in order to address previous findings and guide future use of the scale. The results indicate that the two-factor model is the best fit. Going forward, the FMPS-B’s subfactors “strivings” and “evaluative concerns” may be studied separately. Implications for future research and challenges in bifactor modeling are discussed.

## Introduction

Perfectionism is reportedly on the rise both in the United States and Europe and receiving increasing attention worldwide (Curran and Hill, 2019; Smith et al., 2019). In order to research this phenomenon, it is crucial to have reliable, valid, and effective tools to measure perfectionism. It is important to note that the slight differences in which we operationalize and measure perfectionism today can result in research on different but related constructs (Hewitt et al., 2003; Shafran et al., 2003). Although there currently is no guiding definition of perfectionism, it is often defined as consisting of unrealistically high expectations and overly critical self-evaluations (Frost et al., 1990). Researchers also suggest perfectionism may be a transdiagnostic process, central to increasing individuals’ vulnerability to and maintenance of serious mental health problems and an important predictor of treatment outcome (Egan et al., 2011). The concept “perfectionism” has been around for a long time both as a layman term and in the literature and has been repeatedly reconceptualized as a unidimensional construct, two-dimensional construct, or multidimensional construct.

In the early 1990s, two identically named scales were developed by Frost et al., 1990 [Multidimensional Perfectionism Scale (FMPS)], and closely followed by Hewitt and Flett (1991) [Multidimensional Perfectionism Scale (MPS)]. Frost et al. (1990) defined perfectionism as “setting of excessively high standards for performance accompanied by overly critical self-evaluation” and first identified six dimensions in the 35-item scale: “concern over mistakes,” “personal standards,” “parental expectations,” “parental criticism,” “doubts about actions,” and “organization.” However, because “organizations” loose correlations to the other subscales, the authors recommend that it not be included in calculating total scores (Frost et al., 1990).

Both the FMPS and MPS provided 30 years of research tracking changes in perfectionism. Authors Curran and Hill (2019) have observed trends in perfectionism between 1989 and 2016 using the MPS and found that young adults are harder on themselves and report more societal pressures and expectations than previous generations. Similarly, Smith et al. (2019) observed in a meta-analysis of studies including both MPS and FMPS that perfectionism has increased the past 25 years. Perfectionism is also receiving increasing attention in Scandinavia where generation Z is colloquially referred to as the “the generation of performance anxiety” (Madsen, 2018). Because of the rise in perfectionism over time and its suggested role in maintaining serious mental health problems, interventions are needed in order to address maladaptive perfectionism (Egan et al., 2011; Curran and Hill, 2019). Both American and Norwegian longitudinal studies report a rise in mental health problems among young adults (Knapstad et al., 2018; Twenge et al., 2019). The 2018 Norwegian Students’ Health and Wellbeing Study found that 29% of students report serious mental health problems compared to 16% only 8 years prior, and 47% of students report they always/usually set very high goals for themselves (Knapstad et al., 2018). The success and implications of decreasing maladaptive perfectionism are largely unknown but could, if proven effective, have important implications for public mental health and treatment outcomes (Egan et al., 2011). Hence, a reliable instrument of perfectionism is needed in order to measure changes in perfectionism, to understand and differentiate between adaptive and maladaptive perfectionism, and to increase our knowledge of how these changes are related to changes in mental health.

Since the development of the first two multidimensional perfectionism scales in the 1990s, the dimensions have been repeatedly psychometrically tested. Through the use of factor analysis, Stoeber and Otto (2006) combined different subscales of different measures, including the FMPS, MPS, Perfectionism Inventory (Hill et al., 2004), Perfectionism Questionnaire (Rhéaume et al., 1995), and Almost Perfect Scale – Revised (Slaney et al., 2001), into two latent dimensions named “perfectionistic strivings” and “perfectionistic concerns.” The FMPS subscales “doubts about actions” and “concerns over mistakes” were included in the dimension coined “perfectionistic concerns,” whereas “perfectionistic strivings” includes the FMPS subscale “personal standards.” The authors argue that the FMPS subscales “organization,” “parental expectation,” and “parental criticism” could be disregarded for conceptualization of “perfectionistic strivings” and “perfectionistic concerns” (Stoeber and Otto, 2006). “Perfectionistic concerns” is often referred to as maladaptive or unhealthy perfectionism in the literature, highlighting its association to a multitude of negative mental health outcomes. Perfectionistic concerns have been linked to anxiety disorders, stress, depression, eating disorders, and obsessive–compulsive disorder (Egan et al., 2011). In contrast, “perfectionistic strivings” more often correlates with positive mental health outcomes (Stoeber and Otto, 2006). However, whether perfectionistic strivings is adaptive is debated. According to Egan et al. (2011), perfectionistic striving is also elevated in clinical samples. Stoeber and Otto (2006) argue striving correlations to positive mental health outcomes become most consistently evident after partialing out the overlap between strivings and evaluative concerns. However, Smith and Saklofske (2017) utilized bifactor modeling and call into question the adaptiveness of strivings because of evidence that specific factor scores for the two dimensions are unreliable and therefore question the practice of removing their general variance, as Stoeber and Otto (2006) suggest. In 2016, FMPS–Brief (FMPS-B) was further developed to represent these two core constructs: evaluative concerns and strivings (Burgess et al., 2016). A notable strength in this study, the support for the FMPS-B, was found utilizing several different samples using a confirmatory factor analysis (CFA), and the authors eliminated items that have historically performed inconsistently, for example, items with cross-loadings (Frost et al., 1990; Burgess et al., 2016).

In summary, because of different factor loadings in different analyses, such as evidence of a two-factor model through CFA and evidence of a stronger general factor in bifactor modeling, the aim of this study is to translate the FMPS-B and examine the psychometric properties of the subfactors, perfectionistic strivings, and concerns. This was accomplished by conducting a pure exploratory bifactor analysis and CFA in a Norwegian sample of university students.

## Materials and Methods

### Participants

The study sample consists of university students (*N* = 383) attending the University of Bergen and Norwegian School of Economics and Business Administration in the western part of Norway. The mean age of the participants was 27 (range = 19–65) years. The sample consists of 20.9% men and 78.9% women. Information on the study was distributed through various university and faculty websites and/or faculty newsletters.

### Materials

#### Frost Multidimensional Perfectionism Scale – Brief

Frost Multidimensional Perfectionism Scale – Brief consists of a total of eight questions, with each subscale comprising four items (Table 1). The suggested subscales are called evaluative concerns and strivings. The items are scored on a Likert scale from 1 (strongly disagree) to 5 (strongly agree) for a minimum total score of 8 and a maximum of 40 and minimum subscale score of 4–20. Higher scores indicate more perfectionistic tendencies. The Cronbach’s α coefficient shows good internal consistency (α = 0.83). The subscale evaluative concerns’ mean was 13.30 (SD = 3.88), and that of the subscale strivings was 13.49 (SD = 3.92) (see Table 2 for descriptive statistics).

**TABLE 1:** FMPS-B subscales and items.

**Evaluative concerns**
1. If I fail at work/school, I am a failure as a person 3. If someone does a task at work/school better than me, then I feel like I failed at the whole task 6. If I do not do well all the time, people will not respect me 8. The fewer mistakes I make, the more people will like me
**Strivings**
2. I set higher goals for myself than most people 4. I have extremely high goals 5. Other people seem to accept lower standards from themselves than I do 7. I expect higher performance in my daily tasks than most people

*Itemized in order of appearance in original scale. Original FMPS items: 9, 12, 13, 19, 24, 25, 30, and 34.*

**TABLE 2:** Descriptive statistics of FMPS-B items.

Item	*N*	Mean	SD	Median	Trimmed	Mad	Min	Max	Skew	Kurtosis	SE
1	383	3.76	1.16	4	3.90	1.48	1	5	–0.96	0.07	0.06
2	383	3.61	1.15	4	3.70	1.48	1	5	–0.59	–0.49	0.06
3	383	3.15	1.26	4	3.18	1.48	1	5	–0.27	–1.14	0.06
4	383	3.37	1.25	4	3.44	1.48	1	5	–0.37	–0.98	0.06
5	383	3.20	1.15	3	3.22	1.48	1	5	–0.19	–0.83	0.06
6	383	3.13	1.27	3	3.16	1.48	1	5	–0.28	–1.13	0.06
7	383	3.36	1.14	4	3.43	1.48	1	5	–0.44	–0.51	0.06
8	383	3.27	1.25	4	3.34	1.48	1	5	–0.39	–0.95	0.06

#### State–Trait Anxiety Inventory

Symptoms of anxiety were measured using the 20-item trait anxiety subscale of the State–Trait Anxiety Inventory (STAI; Spielberger et al., 1983). Respondents indicate general feelings on a Likert scale from 1 (almost never) to 4 (almost always). The scale is validated in a Norwegian sample (Håseth et al., 1990). In our sample, the STAI had a Cronbach’s α = 0.90 (mean = 53.76, SD = 11.13).

#### Major Depression Inventory

The 13-item Major Depression Inventory (MDI) measures symptoms of depression on a Likert scale from 1 (not at all) to 6 (all of the time) (Bech et al., 2001). Respondents are asked to indicate the presence of symptoms over the last 2 weeks. Two items consist of pairs, in which the highest scores were included for statistical analysis. The scale is validated in a Danish clinical sample (Olsen et al., 2003). In this sample, the MDI had a Cronbach’s α = 0.88 (mean = 21.44, SD = 10.60).

### Procedure

The study was approved by the Norwegian Regional Committees for Medical and Health Research Ethics-North 2015/2211. We asked two universities and five faculties to aid in recruiting participants online. These institutions distributed information on the study on official faculty/university websites, Facebook pages, newsletters, and by e-mail. Students were provided with brief information on the study by their representative faculties followed by a link. The link immediately provided students with informed consent forms in SurveyXact (2018). In order to move past the informed consent form and fill out the questionnaire, students had to confirm they had read the informed consent and wanted to participate in the study. The data were collected at the beginning of three semesters from the spring of 2018 to spring of 2019. The numbers of questionnaires distributed by e-mail each semester were 827, 525, and 1,029, respectively. The link was active for 2–7 days, upon which it was deactivated, and students were thanked for their interest in participating and provided with information on participation in future semesters, until the last data collection. As an incentive for participation, students who filled out the questionnaire entered our lottery to win two movie tickets. The first author, who is fluent in both English and Norwegian, translated the FMPS. The scale was then back-translated by a second bilingual individual in order to confirm that the translation reflected the measure’s original intended meaning as outlined by the World Health Organization’s translation process guidelines for forward translation and back-translation (World Health Organization [WHO], 2020).

### Statistical Analysis

Four hundred twenty-three participants began filling out the survey; 34 participants were excluded because of no values in the FMPS, leaving a remaining 391 observations. Of these, eight were excluded because of greater than 37.5% missing data. These remaining missing values, which made up 0.98% of the data, were imputed through multiple imputation by chained equations using the mice package in R (Buuren and Groothuis-Oudshoorn, 2011; R Core Team, 2016). Basic descriptive statistics were conducted to evaluate each item for skew and kurtosis, with scores between −2 and +2 considered acceptable indicators of normal distribution (George, 2011). We utilized the Kaiser–Meyer–Olkin (KMO) measure of sampling adequacy and Bartlett test of sphericity in order to inspect whether the data were appropriate for conducting a factor analysis. To evaluate the presence of unidimensionality in the FMPS-B, we conducted a pure exploratory bifactor analysis applying the program FACTOR (Lorenzo-Seva and Ferrando, 2013, 2019). We estimated the closeness to unidimensionality (Ferrando and Lorenzo-Seva, 2017) through values of unidimensional congruence (UniCo) and explained common variance (ECV). UniCo values greater than 0.95 and ECV values greater than 0.85 suggest that the data can be treated as essentially unidimensional. Furthermore, we applied a CFA in order to evaluate the two-factor structure of the eight-item FMPS-B, as suggested by Burgess et al. (2016). The CFA analysis was conducted with robust maximum likelihood estimation, using the “lavaan” package in R (Rosseel, 2011; R Core Team, 2016). For comparison, we applied the same fit criteria as Burgess et al. (2016). Thus, the comparative fit index (CFI; Bentler, 1990) and the root mean square error of approximation (RMSEA; Steiger, 1990) were used as indicators of model fit, with CFI values greater than 0.90 and 0.95 and RMSEA values less than 0.10 and 0.05, indicating good and excellent fit, respectively (Kline, 2005).

Informed by theory and previous research, we evaluated the convergent validity of the FMPS-B by analyzing both subscales’ Pearson correlations to measures of depression (MDI; Bech et al., 2001) and anxiety (STAI; Spielberger et al., 1983). Of the original 383 participants, 355 completed both the MDI and STAI and were included in this analysis. Historically, evaluative concerns are expected to correlate statistically significantly to both anxiety and depression. The subscale strivings are expected to have a weaker correlation or none.

## Results

The KMO measure of sampling adequacy suggests that data seem appropriate for factor analysis [KMO = 0.80, confidence interval (CI) = 0.78–0.85]. Bartlett test of sphericity suggests that there is sufficient significant correlation in the data for factor analysis [χ^2^(28) = 1163.83, *p* < 0.001].

The pure exploratory bifactor analysis suggests that the FMPS-B does not perform as a unidimensional instrument. The overall unidimensional congruence in the FMPS-B is less than 0.95 (UniCo = 0.66, BC bootstrap 95% CI = 0.54–0.73). The value of ECV is less than 0.85 (ECV = 0.46, BC bootstrap 95% CI = 0.03–0.72).

However, on an item level, items 3 and 7 (respectively) have item i-UniCo values greater than 0.95. No items have i-ECV values greater than 0.85 (Table 3). In addition, all items load significantly on the G factor (0.30) in the bifactor model rotated loading matrix, and item 3 only loads significantly on the G factor and not on any of the subfactors (Table 4).

**TABLE 3:** Exploratory bifactor model: item-level closeness to unidimensionality.

Item	I-UniCo	BC bootstrap 95% CI	I-ECV	BC bootstrap 95% CI
Item 1	0.77	(0.16–0.99)	0.52	(0.01–3.15)
Item 2	0.22	(0.00–0.63)	0.18	(0.00–1.95)
Item 3	**0.97**	(0.37–1.00)	0.81	(0.01–1.47)
Item 4	0.60	(0.04–0.98)	0.41	(0.01–6.46)
Item 5	0.80	(0.19–1.00)	0.57	(0.01–3.09)
Item 6	0.68	(0.25–0.99)	0.46	(0.02–4.20)
Item 7	**0.95**	(0.67–1.00)	0.75	(0.36–4.83)
Item 8	0.26	(0.01–0.78)	0.20	(0.00–2.22)

*A value of UniCo (Unidimensional Congruence) and I-Unico (Item Unidimensional Congruence) larger than 0.95 suggests that data can be treated as essentially unidimensional. A value of ECV (explained common variance) and I-ECV (item explained common variance) larger than 0.85 suggests that data can be treated as essentially unidimensional. UniCo and ECV loading greater than 0.95 and 0.85, respectively, are in bold font.*

**TABLE 4:** Rotated loading matrix in exploratory bifactor model.

Item	Factor strivings	Factor evaluative concerns	*G* factor
1	–0.12	**0.44**	**0**.**48**
2	**0.85**	0.20	**0**.**40**
3	–0.05	0.27	**0**.**57**
4	**0.61**	0.26	**0**.**53**
5	**0.51**	–0.01	**0**.**59**
6	–0.14	**0.62**	**0**.**59**
7	**0.44**	–0.04	**0**.**77**
8	–0.11	**0.75**	**0**.**39**

*Factor loading greater than 0.30 are in bold font.*

Overall, the pure bifactor exploratory analysis indicates that the unidimensional model is not a good fit despite some unidimensionality on item level. The CFA indicates that the two-factor model suggested by Burgess et al. (2016) has a good to excellent fit (CFI = 0.94; RMSEA index = 0.09, standardized root mean square residual (SRMR) = 0.07) (Table 5).

**TABLE 5:** Two-factor loading in confirmatory factor analysis.

	Estimate	Std. err	*z* value	*P*(>|*z*|)	Std. lv	Std. all

Latent variables						
**Evaluative concerns**						
mps 1	1.00			0.74	0.64	
mps 3	0.95	0.11	8.97	0.00	0.70	0.56
mps 6	1.40	0.12	11.31	0.00	1.03	0.82
mps 8	1.26	0.12	10.99	0.00	0.93	0.74
**Strivings**						
mps 2	1.00			0.94	0.82	
mps 4	1.05	0.07	15.52	0.00	0.98	0.79
mps 5	0.87	0.06	13.88	0.00	0.81	0.71
mps 7	0.92	0.06	14.93	0.00	0.86	0.76

**Covariances**						

**Evaluative concerns**						
Strivings	0.23	0.05	4.93	0.00	0.34	0.34

Evaluative concerns correlate significantly to both measures of anxiety [*r*(353) = 0.53, *p* < 0.01] and depression [*r*(353) = 0.42, *p* < 0.01], whereas striving has a weak correlation to anxiety [*r*(353) = 0.14, *p* < 0.01] and does not correlate significantly to depression. This is also consistent with previous findings (Table 6).

**TABLE 6:** Correlations of factor strivings and evaluative concerns with measures of anxiety and depression.

	Factor strivings	Factor evaluative concerns
Anxiety	0.14*	0.53*
Depression	0.06	0.42*

*p < 0.01.

## Discussion

The primary aim of this study was to evaluate the fit of the unidimensional and two-factor model of the FMPS-B using a pure bifactor analysis and a CFA in a Norwegian sample due to previous mixed findings. Findings from the exploratory bifactor analysis indicate that two of the eight items perform unidimensionally, and all items from the FMPS-B load on a general factor, as previously indicated by Smith and Saklofske (2017). However, the general factor is weak in comparison to the specific subfactors. Thus, the results of the pure exploratory bifactor analysis do not support perfectionism as measured by the FMPS-B as unidimensional, that is, the use of the total sum score of the FMPS-B. Smith and Saklofske (2017) also conducted both CFA and bifactor analysis on three combined samples of the MPS, original FMPS, and APS-R. The authors compared goodness of fit and chose the bifactor with a strong general factor model as the best representation of perfectionism (Smith and Saklofske, 2017). However, a concern in bifactor modeling is “overfitting” due to capturing of unwanted noise and bifactor models’ propensity to fit even random patterns (Bonifay et al., 2017). As a result, authors warn not to adopt models based primarily on which fit better (Murray and Johnson, 2013; Bornovalova et al., 2020). In addition, one would expect that pooling items from several different instruments together in one analysis increases the G factor relative to the subgroups. This is because there will be several items that overlap in content and also a propensity of item cross-loading to different subgroups when analyzing multiples scales simultaneously. However, the bifactor analysis forces the items into orthogonal solutions/subgroups, relative to each other. Thus, in a situation with item cross-loadings, the G factor will be stronger because of this misfit between bifactor model restrictions and item variance (Bornovalova et al., 2020). In our view, this is one explanation as to why Smith and Saklofske (2017) find a strong G factor, whereas we do not. As stated earlier, when the FMPS-B was developed, items showing a pattern of cross loadings were explicitly removed. Thus, in our situation, there is no conflict between the orthogonal bifactor restriction and the item variances within the scale. As such, our results replicate the good to excellent fit Burgess et al. (2016) found for their two-factor model consisting of evaluative concerns and strivings.

At the item level, we observed that one item (item 3) loaded only on the G factor and not on any subgroups. In order to explain this, we must take a closer look at the item and factor content. The evaluative concerns subfactor consists of a total of four items. Three of these questions, item 1, 6, and 8, measure the extent to which an individual generalizes failure/mistakes to their social or self-worth, that is, “If I fail at work/school, I am a failure as a person.” Two of these items, 6 and 8, are formed to measure the same desire, to avoid mistakes, but distinguish themselves from each other by fear-based versus reward motivation. Thus, this last item, 3, is thematically different from the other three in that it does not measure the extent to which the individual experiences his/her worth to be affected by lack of perfection: “If someone does a task at work/school better than me, then I feel like I failed at the whole task.” Instead, the item generalizes lesser achievement to failure and evaluates an interesting competitive or comparative aspect of perfection motivation. This is the only item that in our analysis loads significantly only on the general factor and not the specific factors.

While the FMPS-B performs well as a two-factor measurement of the subfactors strivings and evaluative concerns in perfectionism, there are two items that perform unidimensionally at the item level. These items distinguish themselves in being thematically different from the other subgroup items, in generalizing lesser achievement to failure, and more specific, in comparing ones’ performance in one’s daily tasks (item 30). Shafran et al. (2002), who coined “clinical” perfectionism, argue the multidimensional perfectionism understanding may be too broad and does not reflect the most critical aspects of perfectionism. However, neither of these items deviates from today’s core conceptualization of perfectionism of unrealistically high expectations and negative self-evaluations.

The FMPS-B shows good internal consistency in a Norwegian sample. The subscale strivings performs consistently with previous samples, whereas evaluative concerns’ mean is higher (mean = 13.30, SD = 3.88) than reported in Burgess et al. (2016) samples (community mean = 9.99, SD = 4.02; clinical mean = 11.89, SD = 4.10). This slight difference may indicate, as previous longitudinal studies have reported, that perfectionism is increasing among young adults. However, it is impossible to draw conclusions on whether these differences in mean evaluative concerns scores from earlier samples reflect an increase over time or differences in populations. The FMPS-B also exhibits good convergent validity in this Norwegian sample. The subscale evaluative concerns correlates significantly to symptoms of anxiety and depression, as expected from previous literature. Research highlights evaluative concerns’ maladaptive role in mental health, whereas the role of strivings is still debated. Striving is more often linked to positive mental health; however, striving is also found to be elevated in clinical samples (Egan et al., 2011). In this sample, strivings had only a weak correlation to anxiety.

## Strengths and Limitations

Findings from this study are limited to the FMPS-B. Other limitations include that the data consist entirely of self-report. Participants consist of a large student population in the Western part of Norway, this homogeneity may limit generalizability to other populations. The strengths of this study are a large sample for sufficient statistical power, sound methodology, including an analysis of two different statistical approaches that have previously resulted in inconsistent findings in the field. In addition, the use of a single scale allows for greater generalizability of our findings to the use of FMPS-B and greater usability for future longitudinal outcome studies that require a single, brief, and valid measurement of perfectionism to reduce dropout and test exhaustion when surveying participants repeatedly over time.

## Conclusion

With the influx of research identifying the negative effects and correlations of perfectionism, there is increasing debate in regard to whether perfectionism can be adaptive and simultaneously if perfectionism is unidimensional or not. Most importantly, the field is currently in need of a unifying definition of perfectionism, which can contribute to more collaboration and greater generalizability of this growing field of research (Stoeber, 2018). The bifactor and the CFA taken together overall support the two-factor model, indicating that the FMPS-B lends itself best to studying correlations and changes in evaluative concerns and strivings separately. Future research should employ longitudinal studies to investigate the malleability and adaptability of strivings and evaluative concerns and their mental health correlates. More longitudinal studies on these subfactors would increase our understanding of which factors contribute to the development of mental health problems and treatment resistance in individuals with perfectionism. Pinpointing these areas would have important clinical implications by guiding research to develop more fine-tuned and effective interventions for maladaptive perfectionism.

## Data Availability Statement

The raw data supporting the conclusions of this article will be made available by the authors, without undue reservation.

## Ethics Statement

The studies involving human participants were reviewed and approved by Norwegian Regional Committees for Medical and Health Research Ethics-North. The patients/participants provided their written informed consent to participate in this study.

## Author Contributions

VW, P-EB, and HM contributed to the conception and design of the study. VW collected the data, organized the database, and wrote the first draft of the manuscript. HM performed the statistical analyses with contribution from VW. HM and P-EB wrote the sections of the manuscript. All authors contributed to the manuscript revision, read, and approved the submitted version.

## Conflict of Interest

The authors declare that the research was conducted in the absence of any commercial or financial relationships that could be construed as a potential conflict of interest.
